# Long-Endurance Collaborative Search and Rescue Based on Maritime Unmanned Systems and Deep-Reinforcement Learning †

**DOI:** 10.3390/s25134025

**Published:** 2025-06-27

**Authors:** Pengyan Dong, Jiahong Liu, Hang Tao, Yang Zhao, Zhijie Feng, Hanjiang Luo

**Affiliations:** 1 College of Computer Science and Engineering, Shandong University of Science and Technology, Qingdao 266590, China; dongpengyan@sdust.edu.cn (P.D.); liugua@sdust.edu.cn (J.L.); taohang@sdust.edu.cn (H.T.); zhaoyang@sdust.edu.cn (Y.Z.); 2 School of Information Engineering, Qingdao Binhai University, Qingdao 266555, China; bhfzj@163.com

**Keywords:** maritime search and rescue, maritime unmanned systems, vision sensing, cooperative search, reinforcement learning

## Abstract

Maritime vision sensing can be applied to maritime unmanned systems to perform search and rescue (SAR) missions under complex marine environments, as multiple unmanned aerial vehicles (UAVs) and unmanned surface vehicles (USVs) are able to conduct vision sensing through the air, the water-surface, and underwater. However, in these vision-based maritime SAR systems, collaboration between UAVs and USVs is a critical issue for successful SAR operations. To address this challenge, in this paper, we propose a long-endurance collaborative SAR scheme which exploits the complementary strengths of the maritime unmanned systems. In this scheme, a swarm of UAVs leverages a multi-agent reinforcement-learning (MARL) method and probability maps to perform cooperative first-phase search exploiting UAV’s high altitude and wide field of view of vision sensing. Then, multiple USVs conduct precise real-time second-phase operations by refining the probabilistic map. To deal with the energy constraints of UAVs and perform long-endurance collaborative SAR missions, a multi-USV charging scheduling method is proposed based on MARL to prolong the UAVs’ flight time. Through extensive simulations, the experimental results verified the effectiveness of the proposed scheme and long-endurance search capabilities.

## 1. Introduction

Maritime accidents have risen with the growth of maritime activities such as transportation and resource exploitation. Consequently, maritime search and rescue (SAR) operations play a crucial role in saving lives and protecting property [1,2]. Meanwhile, advancements in unmanned system technology, such as unmanned aerial vehicles (UAVs) and unmanned surface vehicles (USVs), have been used for a variety of maritime activities, including data transmission [3], maritime communications [4,5], and emergency rescue [6]. However, a single type of USV faces challenges in SAR missions. Generally, the traditional USV search approaches obtain target information through sensor equipment, such as cameras and radars, but their perception range is limited due to their low viewing angles. In contrast, UAVs have unique advantages in acquiring information due to the high-altitude viewing angle and wide sensing range. Thus, UAVs can be utilized to design UAV–USV collaborative SAR systems by leveraging their complementary strengths [7].

Although the collaboration search systems of UAVs and USVs have made significant progress in multiple areas, they still face many challenges in information sharing and task collaboration, especially in complex marine conditions or large-scale search missions. Wang et al. [8] studied the visual navigation and control of cooperative unmanned vehicles in maritime SAR missions, and extracted target location information from images captured by UAV, aiming to improve the visual positioning accuracy and computational efficiency of UAVs. However, the real-time performance could be further investigated. Yang et al. [9] used UAVs and USVs to form a collaborative SAR cognitive mobile computing network, and used reinforcement learning to plan search paths and improve communication throughput. However, they only focus on the optimization of communication network and do not analyze the core search problems such as target recognition and search path optimization. Krishna et al. [10] proposed an approach for monitoring collaborative USVs using an UAV. Although this approach improves the efficiency of target observation, it still relies on manual control, and lacks effective collaboration and information sharing between the UAV and the USV, resulting in poor coordination. Xiao et al. [11] proposed an UAV-assisted USV visual navigation approach. However, the lack of cooperation makes it difficult for UAV and USV to flexibly adjust to real-time conditions, which affects the effectiveness of collaborative search missions.

Furthermore, UAV battery energy limitations are also a major bottleneck in collaborative SAR missions. Particularly in long-endurance missions, limited battery capacity will significantly restrict the system’s working time and mission execution efficiency. Meanwhile, performing complex and persistent operations via UAVs may consume significant battery power, such as video streaming and image processing [12]. This significantly reduces the search time and area. To address the energy limitation issue of UAVs, wireless charging technology [13,14] provides a feasible solution. Unmanned ground vehicles (UGVs) typically serve as energy carriers to carry out regular rendezvous with the UAV for long-term air-to-ground charging. For planning the cooperative routing between UAVs and UGVs, Mondal et al. [15] proposed that UAV can be charged on UGV and thus used a heuristic method to determine the location of the UGV charging stations. Yu et al. [16] investigated the routing problem of energy-limited UAVs accessing a set of locations in minimum time. UAVs can recharge on their way either by landing on fixed charging stations or by utilizing UGVs as mobile charging stations. However, this method with a fixed route is not appropriate for complex maritime search scenarios as the marine environment is unpredictable and diverse. Wang et al. [17] proposed a novel air–ground cooperative UAV recharging framework, in which a group of UAVs compete for charging operations within the mission area. However, this centralized approach may lead to unpredictable charging services and disrupted task execution with maritime search scenarios.

Inspired by these pioneering works, this paper leverages the complementary advantages of both UAV and USV, and proposes a cooperative search and rescue scheme for UAVs and USVs based on a multi-agent reinforcement-learning (MARL) approach. Compared with existing methods, this scheme exploits the full potential of the UAVs and USVs and conducts the first-phase cooperative search using the large field of view of UAVs. The second-phase SAR mission is carried out by taking advantage of the high search accuracy and long cruise capability of the USVs to conduct further search and real-time operation on the detected target. It not only improves the search efficiency through MARL method and probability maps, but also prolongs the flight time of UAVs through the multi-USV charging scheduling method, thereby achieving long-endurance cooperative search [18]. To summarize, the main contributions of this paper are listed as follows.

- We propose a multi-UAV cooperative search (ACS) algorithm leveraging MARL and probability map for the first-phase search. Then, we design a second-phase further search (SFS) algorithm for multi-USV by refining the probabilistic map provided the first-phase search of UAVs.
- To deal with the energy constraints of UAVs and perform long-endurance cooperative maritime search operations, we design a multi-USV charging scheduling (SCS) algorithm based on MADDPG and utilize multiple USVs as mobile charging stations to prolong the flight time of UAVs.
- We conduct extensive simulations to evaluate the feasibility and effectiveness of the proposed scheme.

The rest of this paper is organized as follows. In Section 2, we briefly review the relevant literature. Section 3 provides the system model. In Section 4, we describe the overall scheme and design the algorithm. We evaluate the effectiveness of the proposed algorithm in Section 5. Finally, we conclude our work in Section 6.

## 2. Related Work

In maritime search operations, UAVs are often employed to collaborate with USVs due to the flexibility and large field of view of UAVs [7,10]. Dufek et al. [19] presented a marine casualty incident SAR strategy using the USV–UAV system. However, its navigation and control works were completed by operators. Zhang et al. [20] combined the long endurance capability of USVs with the wide-area coverage of UAVs, improving rescue efficiency in flood disasters. However, the USV relies on the global information provided by the UAV for navigation and mission execution, which may lead to difficulties when performing tasks in harsh environments. Whereas, most min-UAVs (powered by lithium-ion or lithium polymer batteries) have a flight time of approximately 90 min [21]. This greatly restricts the extent and duration of their search operation. Notably, increasing the battery capacity of an UAV beyond a certain point can degrade its flight time due to excessive weight. Therefore, designing effective approaches for battery recharge is critical to sustaining the life cycle of UAV flight.

To address these challenges, it is crucial to consider the energy constraints of UAVs, particularly with maritime search operations, to ensure efficient task completion. Currently, the advancement of wireless power transmission (WPT) technology offers a contactless and fully automated wireless charging solution for UAVs, allowing them to recharge during task execution [21]. This contactless charging method can be achieved through radio frequency (RF) [22], enabling the UAV to achieve wireless directional charging near the ground charging station. Chen et al. [23] introduced a WPT technology represented by the resonant beam charging (RBC), which facilitates convenient recharging of UAVs located far from the coastline. These WPT technologies can be integrated into maritime search scenarios.

Due to the ability of multiple agents for collaborate tasks, MARL has become one of the most popular research methods [24]. Zhou et al. [25] proposed a solution of using a charging UAV (CUAV) to charge mission UAVs (MUAVs) through wireless media while optimizing the deployment of MUAVs and then studied the charging scheduling problem of CUAV. Zhu et al. [26] utilized a reinforcement-learning (RL) algorithm to investigate the scheduling problem of CUAVs. However, CUAVs may consume more energy while flying over the sea to counter factors, such as sea breezes, which can reduce the energy reserve. Messaoudi et al. [27] proposed a multi-agent deep Q-network (MADQN) approach that utilizes unmanned ground vehicles (UGVs) to provide a demand-based charging facility for UAVs, which minimizes the average age of information (AoI) in Internet of Things (IoT) devices. However, the existing works on UAV charging mainly focus on the one-to-one charging pattern [28] or the many-to-one charging pattern [29], neither of which is an appropriate solution for the multi-UAV and multi-USV scenarios.

## 3. System Model

**Figure 1:**
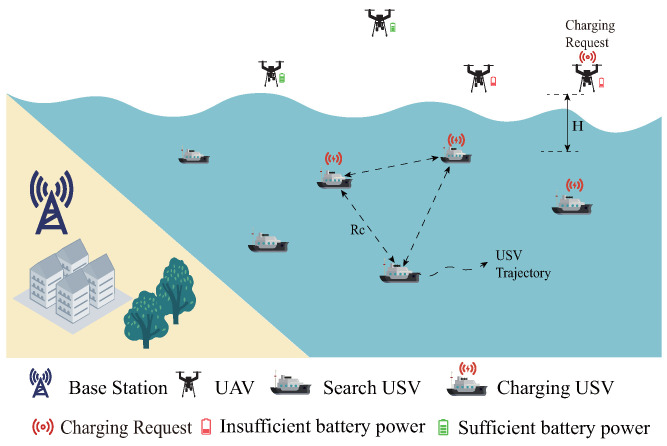
Illustration of the multi-UAV–USV cooperative search system.

As shown in Figure 1, the UAV–USV collaborative search system in the marine environment combines the aerial mobility capability of the UAVs and the advantages of USV’s surface operation to carry out efficient search tasks in the marine environment through cooperation, in which the UAVs are responsible for a quick search task of covering a large area and updating the search probability map. The USVs, as followers, perform a refined search based on the probability map provided by the UAVs. The system consists of *U* USVs and *M* UAVs, and the set of USVs and UAVs is represented as $U=\{U_{i}|i=1,\ldots ,U\}$ and $M=\{M_{i}|i=1,\ldots ,M\}$, respectively. The communication range of each USV is defined as $R_{CU}$, which is a connecting constraint between USVs. If the distance between USVs is greater than $R_{CU}$, they cannot communicate with each other. Moreover, we define the communication range of UAVs as $R_{CM}$. The transmission of information between the USV and the UAV, as well as between the USV and the USV, is achieved through radio communications [30].

### 3.1. Probability Map Model

Due to the uncertainty of the target in a wide range of environments, the modeling method based on probability map has been widely used [31]. We divide the task area into $L_{x}\times W_{y}$ grids, and the central position coordinate of each grid is $g_{k}=\left(x,y\right),\;$$x\;\in \;\left\{1,\ldots ,L_{x}\right\},\;y\in \left\{1,\ldots ,W_{y}\right\},\;k\in \left\{1,\ldots ,L_{x}W_{y}\right\}$. $\theta_{k}=1$ and $\theta_{k}=0$ represent the presence or absence of a target in a grid, respectively. While indicating otherwise possible scenarios, such as the search device failing to obtain the value of $\theta_{k}$,  the probability map remains as it was at the previous moment. As the mission proceeds, the probability map of the mission area is dynamically updated by the UAVs according to predetermined rules [32]. The update equation for the target probability is designed as follows.

(1) $$P_{k}^{t}=\left\{\begin{array}{cc} \frac{dP_{k}^{t-1}}{dP_{k}^{t-1}+f\left(1-P_{k}^{t-1}\right)}, & \mathrm{if}\;\theta_{k}=1\\ \frac{\left(1-d\right)P_{k}^{t-1}}{\left(1-d\right)P_{k}^{t-1}+\left(1-f\right)\left(1-P_{k}^{t-1}\right)}, & \mathrm{if}\;\theta_{k}=0\\ P_{k}^{t-1}, & \mathrm{otherwise}. \end{array}\right.$$

where *d* and *f* are detection probability and false probability, respectively.

Due to the high speed, the UAVs can take the lead in probabilistic detection of the mission area, i.e., UAV $M_{m}$ keeps an individual probability map $P_{m}^{t}\overset{▵}{=}\{P_{k}^{t}\in \left[0,1\right],\;$$k\in \{1,\ldots ,L_{x}W_{y}\}\}$ at time step *t*. When 0<d<1 and 0<f<1, the relation in (1) is equivalent to

(2) $$\frac{1}{P_{k}^{t}}-1=\left\{\begin{array}{cc} \frac{f}{d}\left(\frac{1}{P_{k}^{t-1}}-1\right), & \mathrm{if}\;\theta_{k}=1\\ \frac{1-f}{1-d}\left(\frac{1}{P_{k}^{t-1}}-1\right), & \mathrm{if}\;\theta_{k}=0\\ \frac{1}{P_{k}^{t-1}}-1, & \mathrm{otherwise}. \end{array}\right.$$

We use a nonlinear transformation $Q_{k}^{t}$ instead of $P_{k}^{t}$ to perform the calculation more effectively.

(3) $$Q_{k}^{t}≜\ln\left(\frac{1}{P_{k}^{t}}-1\right).$$

Then, the relation in (2) is transformed into

(4) $$Q_{k}^{t}=Q_{k}^{t-1}+q_{k}^{t},$$

where

(5) $$q_{k}^{t}≜\left\{\begin{array}{cc} \ln\left(\frac{f}{d}\right), & \mathrm{if}\;\theta_{k}=1\\ \ln\left(\frac{1-f}{1-d}\right), & \mathrm{if}\;\theta_{k}=0\\ 0, & \mathrm{otherwise}. \end{array}\right.$$

Once each UAV updates the probability map according to the observation results, the resultant map is broadcast to the adjacent UAVs for the fusion of information. Let us define the neighbors of UAV $M_{m}$ as $\left\{||p_{j}^{t}-p_{m}^{t}||\leq R_{CM},j\in 1,\ldots ,M,j\neq m\right\}$.

### 3.2. Energy Consumption Model

The movement of the USV is accompanied by its energy consumption. We define $l_{mov}^{t}$ as the traveled distance of the USV $U_{u}$ at time slot *t*, which can be expressed as [33],

(6) $$l_{mov}^{t}=\sqrt{\left|\right|p_{u}^{t}-p_{u}^{t-1}{\left|\right|}^{2}}.$$

Therefore, energy consumption of USV can be written as follows.

(7) $$E_{cost}^{u}\left(t\right)=\alpha \times l_{mov}^{t}+R_{es},$$

where α is a coefficient and $R_{es}$ is the resistance of the USV to travel under maritime conditions. Therefore, the energy consumption of *U* USVs until time *T* is given by

(8) $$E_{U}=\sum_{t=1}^{T}\sum_{u=1}^{U}E_{cost}^{u}\left(t\right).$$

The energy consumption of UAVs is mainly divided into two categories: communication energy consumption and flight energy consumption. Since the communication energy consumption is much lower than the flight energy consumption, the communication power $P_{com}$ is fixed to simplify the system. The flight power of the UAV at a uniform speed *v* is as follows [34].

(9) $$\begin{array}{cc} P_{fly}\left(v\right) & =P_{0}\left(1+\frac{3v^{2}}{U_{tip}^{2}}\right)+P_{y}{\left(\sqrt{1+\frac{v^{4}}{4v_{0}^{4}}}-\frac{v^{2}}{2v_{0}^{2}}\right)}^{1/2}+\frac{1}{2}d_{0}\rho_{a}S_{R}A_{s}v^{3}, \end{array}$$

where $P_{0}$ and $P_{y}$ represent the blade profile power and induced power in hovering condition, respectively. $U_{tip}$ denotes the tip speed of the rotor blades, and $v_{0}$ is the mean rotor-induced velocity in the hovering state. $d_{0}$ and $S_{R}$ represent the fuselage drag ratio and rotor solidity, respectively, while $\rho_{a}$ and $A_{s}$ denote the air density and rotor disc area, respectively. Therefore, the energy consumption of UAV $M_{m}$ up to time slot *t* is expressed as

(10) $$E_{m}=\int_{0}^{t}\left(P_{com}+P_{fly}\left(v\right)\right)dt.$$

### 3.3. Wireless Charging Model

We adopt electromagnetic induction wireless charging technology in this study [35]. When an UAV’s battery is lower than the preset threshold, it sends out an energy supply request. After finishing a response of an USV, the UAV performs landing through GPS or visual integration positioning systems. After successful landing of the UAV, the USV battery recharging system converts the input voltage into a high-frequency alternating current, driving the transmitting coil to generate an alternating magnetic field. The UAV’s receiving coil captures the magnetic field energy and converts it into direct current, which is then adjusted to a battery-adaptive voltage, enabling efficient charging.

Based on the input voltage and working current of the power supply, calculate the input power before voltage boost as $P_{in}=V_{in}\times I_{in}$. Then, estimate the output power after voltage boost by conversion efficiency is $P_{boost}=P_{in}\times \eta_{boost}$. After the magnetic field energy captured by the receiving coil is rectified, the output DC power is $P_{recv}=P_{boost}\times \eta_{coupling}\times \eta_{rect}$, where $\eta_{coupling}$ is the magnetic coupling efficiency and $\eta_{rect}$ is the rectification efficiency.

According to the charging time and the receiving power, the calculated charging energy is $E_{har}^{t}=\int_{0}^{t}P_{recv}dt$. We assume that the maximum energy capacity of each UAV is $E_{max}$. Therefore, the residual energy of the UAV $M_{m}$ is expressed as follows.

(11) $$E_{re_{m}}^{t}=\left\{\begin{array}{cc} E_{max}, & \mathrm{if}\;E_{re_{m}}^{t-1}+E_{har}^{t}\geq E_{max},\\ E_{re_{m}}^{t-1}+E_{har}^{t}, & \mathrm{otherwise}. \end{array}\right.$$

where the energy of UAV $M_{m}$ at time *t* satisfies the $E_{th}\leq E_{re_{m}}^{t}\leq E_{max}$, where $E_{th}$ is the minimum reserved battery energy to prolong the battery lifetime [36].

Meanwhile, we define the charging urgency of UAV $M_{m}$ to reflect the importance of the UAVs charging sequence, which can be calculated as

(12) $$\zeta_{m}\left(t\right)=1-\frac{E_{re_{m}}^{t}-E_{th}}{E_{max}}.$$

If the remaining energy of the current UAV is low, it has a high charging emergency, and hence the USV gives priority service to the UAV with a high charging emergency.

## 4. The Proposed Scheme

In this section, we first briefly describe the framework of long-endurance collaborative SAR scheme. Then, we provide the ACS and SFS algorithm designs. Finally, we present the multi-USV charging scheduling algorithm.

### 4.1. General Description

In the search mission, the air-mobility capability of the UAV is combined with the advantage of the surface USV. The UAVs are responsible for quickly covering a large area and updating the search probability map for the first-phase search, while the USVs carry out second-phase SAR missions according to the probability map provided by the UAVs. To solve the energy limitation problem of UAVs, multiple USVs are dedicated to providing charging service for UAVs. Due to the continuous changes in the position and residual energy of the UAVs, the USVs should constantly interact with the nearby USVs, optimize the scheduling process, and ensure that the UAVs get sufficient energy to perform long-endurance SAR missions.

### 4.2. Search Algorithms of Multi-UAV and Multi-USV

#### 4.2.1. ACS Algorithm

With its fast moving speed and wide field of view, an UAV is able to quickly cover large mission areas for extensive target detection. After updating the probability map according to the observation results, each UAV broadcasts the information to its neighbors for probability map fusion. The UAV records the number of target detections N(+) and the false detections N(−) for each cell. When $\theta_{k}=1$ holds, N(+) is increased by 1, otherwise, it remains unaltered. Conversely, when $\theta_{k}=0$ holds, N(−) is incremented by 1, otherwise, it also remains unaltered. These target detections and the false detections numbers are used as interactive information between UAVs [37]. N(+) and N(−) of UAV $M_{m}$ at time slot *t* after sufficient fusion of information can be derived as

(13) $$\left\{\begin{array}{c} N_{m,t}\left(+\right)=\max\left(N_{m,t}\left(+\right),\max N_{j,t}\left(+\right)\right),\;j\in Z_{m},\\ N_{m,t}\left(-\right)=\max\left(N_{m,t}\left(-\right),\max N_{j,t}\left(-\right)\right),\;j\in Z_{m}. \end{array}\right.$$

where $Z_{m}$ is the neighbor set of the UAV. Finally, it is obtained that the probability of target existence in the cell $g_{k}$ of the time step *t* after the fusion information is

(14) $$Q_{k}^{t}=N_{m,t}\left(+\right)\ln\frac{f}{d}+N_{m,t}\left(-\right)\ln\frac{1-f}{1-d}.$$

In this subsection, the multi-actor-attention-critic (MAAC) algorithm [38] is adopted for the multi-UAV collaboration search process. Each UAV independently executes an actor-attention-critic model, utilizing a centralized critic equipped with a shared attention mechanism to select pertinent information for each UAV at every time step. This helps the corresponding UAV to extract meaningful information from the observation–action pairs of other UAVs to construct the input for its critic. This approach addresses the scalability issue and aids UAVs in selectively finding important environmental information while ignoring irrelevant data. We model the search process of each UAV as a partially observable Markov decision process (POMDP), represented by the tuple (S,O,A,P,R) as follows.

- State space S: To search the task area, each UAV searches the target and observes the state information of other UAVs, where $s_{i}$ is the state space of UAV *i*, i.e.,
  (15) $$s_{i}=\left\{P_{M},P_{m}^{t},E_{m}\right\},$$
  where $P_{M}$ represents the set of location information of all UAVs, $P_{m}^{t}$ indicates the probability map information, and $E_{m}$ represents the energy consumption of the UAV.
- Observation space O: O is the set of observed values of all UAVs. $o_{i}$ represents the observations of UAV *i*, including the observed position information of UAV *i*, the position information of other UAVs, and the energy consumption of UAVs. Thus, the observation information can be expressed as
  (16) $$o_{i}=\left\{p_{m}^{i},p_{m}^{\prime },P_{k}^{t},E_{m}^{i}\right\},$$
  where $p_{m}^{i}$ denotes the position coordinates of the UAV *i* and $p_{m}^{\prime }$ represents observed position information of its neighbor UAV. $P_{k}^{t}$ indicates the probability of detecting the grid and $E_{m}^{i}$ is the energy consumption of the UAV *i*.
- Action space A: $A=\{a_{i}|i=1,\ldots ,M\}$ includes all actions that all the UAVs may undertake. In the search activities, the actions to be taken by the UAV include moving direction and moving distance. The action taken by UAV *i* at time *t* is expressed as
  (17) $$a_{i}=\left\{\left(x_{i},y_{i}\right)\right\},$$
  where $\left(x_{i},y_{i}\right)$ is the position of the UAV *i* at the next time slot.
- State Transition Probability $P:S^{t}\times A_{1}^{t}\times A_{2}^{t}\ldots \times A_{M}^{t}\to S^{t+1}$ represents the probability of reaching the next state after executing the action in that state.
- Reward function R: An appropriate reward function can help the UAVs explore better actions. The main objective of the exploration pursued by the UAV is to cover the unexplored area as soon as possible, minimize the energy consumption of the UAVs, and avoid collision with other UAVs. Therefore, the reward function is defined as follows.
  **Target reward:** This reward function encourages the UAV to find the target as soon as possible and mark the location of the target. We set that when $P_{k}^{t}\geq \epsilon$, it means that the UAV has determined the target location, where ϵ is a threshold. The reward that the UAV can get when marking a target location is as follows.
  (18) $$R_{search}=\lambda \sum_{g_{k}\in L_{x}\times W_{y}}1_{P_{k}^{t}\geq \epsilon \;and\;P_{k}^{t-1}<\epsilon },$$
  where λ is a positive constant.
  **Coverage reward:** This reward guides the UAVs to quickly cover the mission area, with fewer repetitive searches, and to cover as much unexplored area as possible. Therefore, the UAV search reward is
  (19) $$R_{cover}=-\kappa \;\cdot \;N_{visit}\left(t\right),$$
  where $N_{visit}\left(t\right)$ is the number of visits to the grid and κ is a penalty coefficient.
  **Collision penalty:** We use a penalty mechanism to guide UAV not to collide with other UAVs, and the collision penalty is [39]
  (20) $$R_{p}=\left\{\begin{array}{cc} -10, & \;dist<D_{min},\\ 0, & \;\mathrm{otherwise}. \end{array}\right.$$
  where $D_{min}$ is the safe distance between UAVs.
  In summary, the whole reward function is derived as
  (21) $$R_{i}=\beta_{1}\;\cdot \;R_{search}+\beta_{2}\;\cdot \;R_{cover}+R_{p},$$
  where $\beta_{1}$ and $\beta_{2}$ are the reward correlation coefficients.

The attention mechanism employs a key-value memory model [38]. In this model, each agent queries other agents for information regarding their observations and actions and then uses this information as input to its critic. Specifically, the Q-function of agent *i* can be expressed as follows.

(22) $$Q_{i}^{\psi }\left(O,A\right)=f_{i}\left(g_{i}\left(o_{i},a_{i}\right),x_{i}\right),$$

where $O=\{o_{1},\ldots ,o_{M}\}$ and $A=\{a_{1},\ldots ,a_{M}\}$ represent the set of observations and the set of actions of all agents, respectively. Both $f_{i}$ and $g_{i}$ represent the MLP layer, where $g_{i}$ is used for embedding operations on the observation-action pair of the agent. $x_{i}$ represents environmental information from other agent observation–action pairs, expressed as

(23) $$x_{i}=\sum_{j\neq i}\alpha_{ij}v_{j}=\sum_{j\neq i}\alpha_{ij}h\left(Vg_{j}\left(o_{j},a_{j}\right)\right),$$

where $\alpha_{ij}$ is the attention weight of agent *i* on agent *j*, and $v_{j}$ is the embedding of the observation–action pair of agent *j*, i.e., $g_{j}$ is first used to code the observation–action pair, and then linear matrix *V* is used for linear transformation, and finally non-linear operation *h* (such as leaky ReLU).

The attention weight of $\alpha_{ij}$ is obtained by comparing the similarity between the embedding vector $g_{i}$ and $g_{j}$ of agent *i* and agent *j*. Since the parameters are shared between the critics of different agents, each critic is trained with a joint loss function, namely

(24) $$L_{Q}\left(\psi \right)=\sum_{i=1}^{M}E_{(o,a,r,o^{\prime })∼D}\left[{\left(Q_{i}^{\psi }\left(O,A\right)-y_{i}\right)}^{2}\right],$$

where

(25) $$y_{i}=r_{i}+\gamma E_{a^{\prime }∼\pi_{\bar{\eta }}\left(o^{\prime }\right)}[Q_{i}^{\bar{\psi }}\left(O^{\prime },A^{\prime }\right)-\alpha log\left(\pi_{{\bar{\eta }}_{i}}\left(a_{i}^{\prime }|o_{i}^{\prime }\right)\right].$$

The actor policy of each agent is updated by the following formula.

(26) $$\begin{array}{cc} \; & \nabla_{\eta_{i}}J\left(\pi_{\eta }\right)=E_{o∼D,a∼\pi }[\nabla_{\eta_{i}}log\left(\pi_{\eta_{i}}\left(a_{i}|o_{i}\right)\right)\left(\alpha log\left(\pi_{\eta_{i}}\left(a_{i}|o_{i}\right)\right)-Q_{i}^{\psi }\left(O,A\right)+b\left(O,A_{\setminus i}\right)\right], \end{array}$$

where $b(O,A_{\setminus i})$ is the multi-agent baseline. The brief description of this process is shown in Algorithm 1. We first initialize the network parameters and the experience replay buffer D. According to the initial position of the UAV, the observed state of the environment is obtained. Then, the UAV selects actions through the actor network and performs the actions according to Equation (26) to obtain the reward of environmental feedback. Subsequently, the algorithm stores the current state, action, reward, and transfer information as transformation tuples in the experience replay buffer, thereby improving the utilization efficiency of the data and reducing the correlation among samples. In each training iteration, a small batch of transformation tuples is randomly sampled from the buffer, and the two critic networks are updated according to Equations (24) and (25). Finally, update the parameters of the target network to complete the policy optimization within the round and prepare for the next round of training.
**Algorithm 1** The proposed ACS Algorithm.1:Initialize the actor and critic networks of each UAV.2:Initialize target networks for each UAV.3:Initialize replay buffer D.4:$T_{update}\leftarrow 0$.5:**for** episode=1→E **do**6:     Reset environments and get initial $o_{i}$ for each UAV *i*.7:     **for** t=1→T **do**8:           Select actions $a_{i}∼\pi_{i}\left(\;\cdot \;|\eta_{i}\right)$ for each UAV *i* in each environment *e*.9:           Send actions to all parallel environments and get ${o^{\prime }}_{i},{r^{\prime }}_{i}$ for all agents.10:          Store transition for all environments in D.11:           $T_{update}=T_{update}+E$12:           **if** $T_{update}\geq$ min steps per update **then**13:                 **for** j=1,…,num critic updates **do**14:                       Sample a mini-batch from D.15:                       Update the critic network according to (24) and (25).16:                 **end for**17:                 **for** j=1,…,num policy updates **do**18:                       Sample $m\times (o_{1,\ldots ,M})∼D$.19:                       Update the actor network according to (26).20:                 **end for**21:                 Update target parameters: $\bar{\psi }=\tau \bar{\psi }+\left(1-\tau \right)\psi$; $\bar{\eta }=\tau \bar{\eta }+\left(1-\tau \right)\eta$.22:                 $T_{update}\leftarrow 0$.23:           **end if**24:      **end for**25:**end for**

#### 4.2.2. SFS Algorithm

In maritime search missions, USVs serve as critical executors for precision search operations, leveraging their high-resolution sensor systems and proximity operation advantages to function as key platforms for target detection in refined search scenarios. To optimize search efficiency, USVs must dynamically adjust their search strategies based on target probability maps provided by UAVs, enabling precise coverage of high-probability regions. This paper proposes a cooperative search method based on genetic algorithm (GA) [40], designed to optimize USV navigation paths in complex environments, ensuring maximal coverage of high-probability areas while simultaneously minimizing search path length.

The USV first preprocesses the acquired UAV probability map to identify high-probability target points exceeding threshold ϵ, subsequently constructing a distance matrix $D\in R^{\left(U+N_{s}\right)\times N_{s}}$ to characterize the spatial relationships between USVs and waypoints as well as inter-waypoint connections, where *U* denotes the number of USVs and $N_{s}$ represents the quantity of waypoints, where each matrix element D(i,j) is computed via the Euclidean distance formula,

(27) $$D\left(i,j\right)=\sqrt{{\left(x_{i}-x_{j}\right)}^{2}+{\left(y_{i}-y_{j}\right)}^{2}}.$$

During the initialization phase, the system randomly generates an initial population comprising *N* feasible paths, with each path consisting of multiple interconnected sub-paths. The core of the GA lies in its composite fitness function, mathematically formulated as follows.

(28) $$F=\sum_{j=1}^{K}\left(\frac{\sum_{k=1}^{L_{j}-1}D\left(n_{k},n_{k+1}\right)}{v_{s}}+t_{r}\times L_{j}\right)$$

where *K* denotes the number of sub-paths, $L_{j}$ represents the node count of the *j*-th sub-path, $v_{s}$ indicates the cruising speed of the USV, $D(n_{k},n_{k+1})$ indicates the distance between adjacent nodes in the path, and $t_{r}$ signifies the search execution time required at each path node.

During the evolutionary process, the algorithm employs improved genetic operators for path optimization. In the selection phase, the roulette wheel strategy based on fitness is employed. For the crossover operation, the ordered crossover method is utilized, and the crossover probability determines the exchange of parent path fragments, and the fine-grained search algorithm for a USV is described in Algorithm 2.
**Algorithm 2** The proposed SFS Algorithm.**Input:** Target probability map $P_{m}$ generated by UAV, Population size *N*, Maximum generations MaxGen,Crossover probability $P_{c}$, Mutation probability $P_{m}$.**Output:** Optimal search path and target location.1:Initialize population with *N* random search paths.2:USVs obtain the target probability map $P_{m}$ from UAV and screens the target points.3:Construct distance matrix *D* by computing Euclidean distances between points.4:**for** generation=1 to MaxGen **do**5:      **for** each search path $Path_{i}$ in population **do**6:            Calculate fitness value using Equation (28).7:            Record fitness score.8:      **end for**9:      Select elite paths as parents using roulette wheel selection.10:     Perform crossover operation on parent paths with probability $P_{c}$.11:     Perform mutation operation with probability $P_{m}$.12:     Preserve historically best individual.13:     Update population.14:     **if** the best fitness has not improved for δ consecutive generations **then**15:            **break**16:      **end if**17:**end for**18:Select the path with the highest fitness value as the optimal search path.19:Perform 2-opt local optimization on optimal path.20:**return** Optimal path and target locations.

### 4.3. SCS Algorithm

To deal with the energy constraint issue of UAVs and perform long-endurance maritime search missions, we develop a multi-USV charging scheduling algorithm to prolong the flight time of UAVs in this subsection. We deploy *N* USVs as mobile charging stations specifically to provide wireless charging services for UAVs. When an UAV battery is low, a charging request will be sent to the USVs. Then, the allocated USV plans a path according to the remaining energy level of the UAV and minimizes the moving distance of the USV. Therefore, we propose an USV charging scheduling algorithm based on the multi-agent deep deterministic policy gradient (MADDPG) [41] method. MADDPG is an extension of the deep deterministic policy gradient (DDPG) algorithm by using a centralized training and distributed execution (CTDE) method to use global information in the training process, which is not visible to a single agent. On the other hand, each agent pursues its operations based only on local information. At each time slot t∈T, the USV tracks the current state of the environment ${\tilde{s}}_{i}\left(t\right)$ and pursues action ${\tilde{a}}_{i}\left(t\right)$ according to the specified policy $\pi_{i}\left({\tilde{s}}_{i}\left(t\right)\right)$. Then, environment status ${\tilde{s}}_{i}\left(t+1\right)$ is updated and the agent receives reward ${\tilde{r}}_{i}\left(t\right)$. We model the motion of USV as a partially observed Markov decision process (POMDP) represented by the quintuple $\left(\tilde{S},\tilde{O},\tilde{A},\tilde{P},\tilde{R}\right)$ as follows.

- State Space: $\tilde{S}=\left\{\tilde{s_{1}},\tilde{s_{2}},\ldots ,\tilde{s_{N}}\right\}$ is the global environment information in the system, including the position coordinates of the USV and UAV, the energy level and the current working state of the USV, which is represented as a binary variable $\Psi_{i,m}\left(t\right)$. If $\Psi_{i,m}\left(t\right)$ is set to 1, it indicates that the USV is engaged in the charging process; otherwise, $\Psi_{i,m}\left(t\right)$ equals 0. We use ${\tilde{s}}_{i}$ to represent the state of USV *i* at time slot *t*, i.e.,
  (29) $${\tilde{s}}_{i}=\left\{p_{u}^{i},p_{m},E_{cost}^{i},\Psi_{i,m}\left(t\right)\right\}.$$
- Observation Space: $\tilde{O}$ is the set of observations for all USVs. In a multi-agent system, each USV determines the action based on its current state as well as the current state of its nearby USVs. The observed values include the location information of itself and its neighbors, the location information and the charging urgency of the UAV. The observation space of USV *i* is
  (30) $${\tilde{o}}_{i}=\left\{p_{u}^{i},p_{u}^{\prime },p_{m},\zeta_{m},E_{cost}^{i}\right\},$$
  where $p_{u}^{\prime }$ indicates the position coordinates of the neighbor USV and $E_{cost}^{i}$ represents the energy consumption of USV *i*.
- Action Space: $\tilde{A}$ contains all actions that all USVs may take during the course of exploration, including direction and distance of movement. The action space of USV *i* is represented as
  (31) $${\tilde{a}}_{i}=\left\{\phi_{i}\left(t\right),d_{i}\left(t\right)\right\},$$
  where $\phi_{i}\left(t\right)$ is the movement direction of USV *i* at time slot *t* and $d_{i}\left(t\right)$ is the distance traveled.
- State Transition Probability $\tilde{P}$: This describes the probability that the system transits to another state after performing an action in a state.
- Reward function: $\tilde{S_{i}^{t}}\times \tilde{A_{i}^{t}}\to \tilde{R_{i}^{t+1}}$ denotes the reward of USV *i* at time slot *t* given that the agent observes a system state $\tilde{S_{i}^{t}}$ and takes an action $\tilde{A_{i}^{t}}$. The objective of the USVs is to learn the optimal strategy $\tilde{\pi^{*}}$, which is to maximize the cumulative reward while interacting with the environment. Therefore, we design a reward function based on the local information of each agent as well as the collaborative information to incentivize the USVs to search the target and maintain the UAV battery level while minimizing the energy consumption due to the movement.
  Therefore, the reward function of USV *i* at time *t* is expressed as
  (32) $$\tilde{R_{i}^{t}}=Re_{i}^{t}\times Rc_{i}^{t}+R_{l}+R_{d},$$
  where $Re_{i}^{t}$ represents the energy consumption of USV *i* for executing the corresponding task. $Rc_{i}^{t}$ represents a reward that the USV receives when it charges an UAV at time *t*. $R_{l}$ and $R_{d}$ are the penalty term for the USV failing to charge the UAV on time as well as for the collision, respectively.
  The energy consumed by each USV is determined by the distance it travels. Our objective is to devise an optimal path that minimizes energy consumption. Therefore, the reward for energy consumption is formulated as
  (33) $$Re_{i}^{t}=\frac{1}{E_{cost}^{i}\left(t\right)}.$$
  The item $Rc_{i}^{t}$ is a reward component indicating the profit obtained by successfully charging an UAV at time slot *t*, which is defined as follows.
  (34) $$Rc_{i}^{t}=\left(E_{har}^{t}+k\right)\times \zeta_{m}\left(t\right),$$
  where $E_{har}^{t}$ is the charging energy to the UAV at time *t* and *k* is a positive coefficient, representing a basic reward that encourages energy charging of the USV regardless of the charging outcome. $\zeta_{m}\left(t\right)$ represents the charging urgency of UAV $M_{m}$. When a USV receives multiple charging requests from UAVs, its charging decision is prioritized based on the charging urgency of each UAV. By assessing the current battery level, the USV determines the charging urgency of UAVs and prioritizes charging service accordingly.
  The $R_{l}$ represents a penalty to an USV if it fails to charge any UAV in time and the remaining battery falls below the E*_th_*. We define $R_{l}$ as
  (35) $$R_{l}=\left\{\begin{array}{cc} -Rc_{i}^{t}, & \;E_{re_{m}}^{t}<E_{th},\\ 0, & \;\mathrm{otherwise}. \end{array}\right.$$
  If there is a collision between USVs, there is a penalty, which we define as
  (36) $$R_{d}=\left\{\begin{array}{cc} -10, & \;d\left(i,j\right)<L_{min},\\ 0, & \;\mathrm{otherwise}. \end{array}\right.$$
  where $L_{min}$ represents the safe distance between USVs.

In the MADDPG-based charging scheduling algorithm, each agent *i* maintains its own actor network $\mu_{\theta_{i}}$ and critic network $Q_{\omega_{i}}$, whose network parameters are $\theta_{i}$ and $\omega_{i}$, respectively. The actor network is responsible for interacting with the environment and making action decisions based on the current state. The update strategy for the actor network is as follows.

(37) $$\begin{array}{cc} \; & \nabla_{\theta_{i}}J\left(\mu_{\theta_{i}}\right)=E_{x∼\tilde{D}}\left[\nabla_{\theta_{i}}\mu_{\theta_{i}}\left({\tilde{o}}_{i}\right)\nabla_{{\tilde{a}}_{i}}Q_{\omega_{i}}\left(x,{\tilde{a}}_{1},\ldots ,{\tilde{a}}_{N}\right){|}_{{\tilde{a}}_{i}=\mu_{\theta_{i}}\left({\tilde{o}}_{i}\right)}\right], \end{array}$$

where $\tilde{D}$ is the experience reply buffer which contains past decision of the agents as a tuple $\left(x,a,r,x^{\prime }\right)$ during the course of training.

For the critic networks, we update the loss function of the model by minimizing the squared time difference of the soft Bellman function, which is defined as follows.

(38) $$L\left(\omega_{i}\right)=E_{\tilde{D}}\left[{\left(Q_{\omega_{i}}\left(x,{\tilde{a}}_{1},\ldots ,{\tilde{a}}_{N}\right)-y\right)}^{2}\right],$$

(39) $$y=r_{i}+\gamma Q_{\omega_{i}^{\prime }}\left(x^{\prime },{\tilde{a}}_{1}^{\prime },\ldots ,{\tilde{a}}_{N}^{\prime }\right){|}_{{\tilde{a}}_{j}^{\prime }=\mu_{\theta_{j}^{\prime }}\left({\tilde{o}}_{j}\right)}.$$

The target network is used to avoid training instability. The method of soft update is adopted to update the parameters of the target network, which can be written as

(40) $$\left\{\begin{array}{c} \theta_{i}^{\prime }\leftarrow \tau \theta_{i}+\left(1-\tau \right)\theta_{i}^{\prime },\\ \omega_{i}^{\prime }\leftarrow \tau \omega_{i}+\left(1-\tau \right)\omega_{i}^{\prime }, \end{array}\right.$$

where τ represents the soft update factor. The brief description of this process is shown in Algorithm 3. First, initialize the parameters of the actor network, critic network, and the target network of each agent, and initialize the experience replay buffer $\tilde{D}$. Each agent selects and performs actions based on the current state, interacts with the environment, and obtains corresponding rewards and the next state. Subsequently, the agent stores the tuples of the experienced states, actions, rewards, and the next state in the $\tilde{D}$. Each agent uses the $\tilde{D}$ to update its own critic and actor by sampling mini-batches. After each update, the network parameters of the agent are adjusted to enhance its decision-making ability. When the UAV’s battery power is insufficient, the USV makes a movement decision based on the current state, adjusts the movement direction and distance, then replenish the battery power of the UAV in time, and minimize the movement energy consumption.

The goal of each agent is to maximize its expected cumulative reward as follows.

(41) $$R=\sum_{t=1}^{T}\gamma^{t}\tilde{R_{i}^{t}},$$

where γ∈[0,1] is a discount factor.
**Algorithm 3** The proposed SCS Algorithm.1:Randomly initialize the actor and critic networks of each USV.2:Initialize the target networks of each USV.3:Initialize experience replay buffer $\tilde{D}$.4:**for** episode=1→E **do**5:       Initialize a random process N(t) for explorations.6:       Receive the initial state x.7:       **for** t=1→T **do**8:             For each USV *i*, select action ${\tilde{a}}_{i}=\mu_{\theta_{i}}\left({\tilde{o}}_{i}\right)+N\left(t\right)$.9:             Execute actions $a=({\tilde{a}}_{1},{\tilde{a}}_{2},\ldots ,{\tilde{a}}_{N})$ and observe reward r and new state $x^{\prime }$.10:             Store $\left(x,a,x^{\prime },r\right)$ into the replay buffer $\tilde{D}$.11:             $x\leftarrow x^{\prime }$.12:             **for** USV *i*, i=1,…,N **do**13:                   Sample from $\tilde{D}$ a mini-batch of *S* samples.14:                   Update the actor network according to (37).15:                   Update the critic network according to (38) and (39).16:             **end for**17:             Update the parameters of the target network for each USV *i* according to (40).18:       **end for**19:**end for**

## 5. Experiments

In this section, we first provide a description of the parameter settings, then we conduct simulations to demonstrate the effectiveness of the proposed algorithms.

### 5.1. Simulation Setup

We set the target area as a square area of 10 km × 10 km [42], and the remaining battery capacity of current UAVs follows a uniform distribution of [30%,100%] [17]. The output layer of the critic network is linearly activated, while the actor network uses the tanh function for its output layer to restrict the action range. The hidden layers in all the networks are activated using the ReLU function. For the UAV and USV search algorithms, we perform 20,000 episodes with 300 steps per episode. For the USV charge scheduling algorithm, we perform 20,000 episodes with 100 steps per episode. We implement these algorithms using PyTorch 2.1.2 in python 3.9, which runs on Nvidia 4050 (Santa Clara, CA, USA). The relevant parameters of the algorithm are shown in Table 1.

### 5.2. The Effectiveness of the Proposed Scheme

Based on the Bayesian network framework of probability map model, the uncertainty of the detection system can be modeled by the entropy function [37] $J_{k}^{t}=-P_{k}^{t}logP_{k}^{t}-\left(1-P_{k}^{t}\right)log\left(1-P_{k}^{t}\right)$, where $P_{k}$ is determined by the probability distribution of the detection probability *d* and the false probability *f*. With an initial probability of 0.5, we systematically analyze the effect of different parameter combinations on the uncertainty of the system, as shown in Table 2. The system uncertainty shows a monotonically decreasing relationship with *d* and a monotonically increasing relationship with *f*, which verifies the key role of improving detection performance and suppressing false alarm in reducing system uncertainty. In particular, when both *d* and *f* are taken as 0.5, the uncertainty of the system reaches the maximum value of 1. At this time, the system degenerates into a completely random guessing state. When the detection probability d=0.9 and the false probability f=0.1, the system uncertainty reaches the minimum value of 0.305. This parameter combination shows the optimal performance in all test configurations.

**Figure 2:**
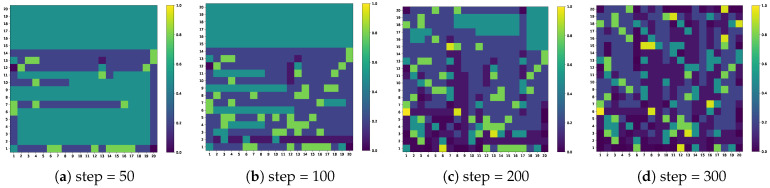
The target probability in the task area changes at different time steps.

To verify the effectiveness of the ACS algorithm, we use heat map analysis to check the change in target probability in different time steps in the task area, as shown in Figure 2. Initially, each grid has a prior probability of 0.5, and the color intensity reflects the target probability value of each grid, where the darker the color means the higher the probability when the target is present. On the contrary, a lighter color means that the possibility of the existence of the target in this area is low. The analysis shows that with the increase in time, the agent’s search region expands gradually, but the target probability in the no-target region decreases significantly. The target probability graph provides important information for the subsequent USV fine-grained search target.

**Figure 3:**
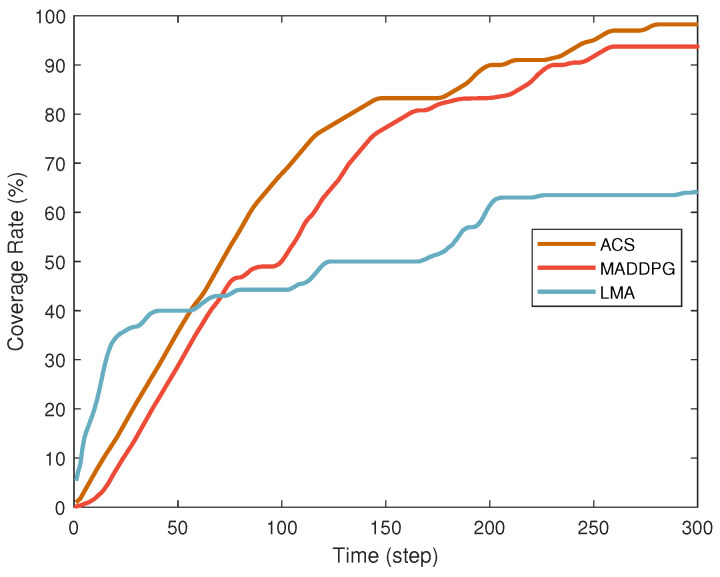
Comparison of coverage under different search algorithms.

Figure 3 compares the performance of different search algorithms in terms of coverage. The lawnmower algorithm (LMA) [1] achieves rapid initial coverage but plateaus later, reaching a final coverage rate of 64.5%, consistent with its fixed-path planning limitations in dynamic environments. In contrast, MADDPG shows slower initial improvement due to agent exploration but demonstrates steady growth over time, reflecting its adaptability in multi-agent collaboration. Our proposed ACS algorithm outperforms both, achieving fast initial coverage and continuous optimization, culminating in a final coverage rate of 98%. Its success stems from efficient collaboration mechanisms and effective balance between exploration and exploitation.

**Figure 4:**
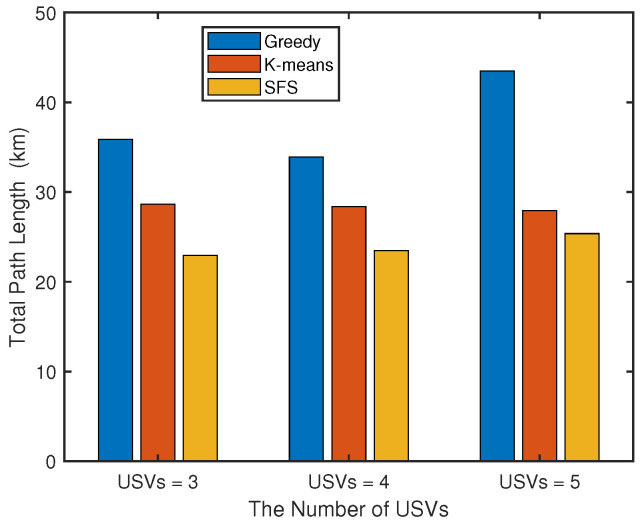
Comparison of path lengths of different algorithms under different number of USVs.

We further compare the total path length performance of the greedy algorithm, K-means clustering algorithm, and SFS algorithm under different number of USVs. As illustrated in Figure 4, the proposed algorithm demonstrates superior path planning performance with significant optimization advantages over conventional methods. The intelligent optimization mechanism of our algorithm, inspired by natural evolutionary processes, effectively overcomes the local optimum trap inherent in greedy algorithms and avoids the adaptability constraints caused by the fixed partitioning pattern of K-means. Particularly noteworthy is the enhanced robustness displayed by our SFS algorithm when handling increased task complexity, attributable to its global optimization characteristics that enable adaptive path allocation. In contrast, traditional methods show varying degrees of performance degradation during scale expansion.

**Figure 5:**
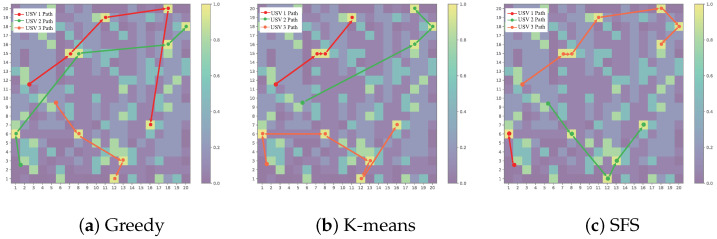
Search path diagram of USVs under different search algorithms.

Figure 5a–c respectively show the comparison of path planning results of the greedy algorithm, K-means clustering algorithm, and the proposed SFS algorithm. It can be seen from the visualization results that the three algorithms show significantly different path characteristics in exploring high-probability regions: the path of the greedy algorithm has obvious path crossing and load imbalance, and the path length of the USV 1 is significantly larger than that of other USVs; the K-means algorithm realizes partition path planning through spatial clustering. Each USV forms a compact path in the specified area, but there are individual detour points at the cluster boundary. The path generated by the SFS algorithm is the smoothest and uniform, and the length difference of each USV path is the smallest and there is no crossover phenomenon, showing the best global optimization capability.

**Figure 6:**
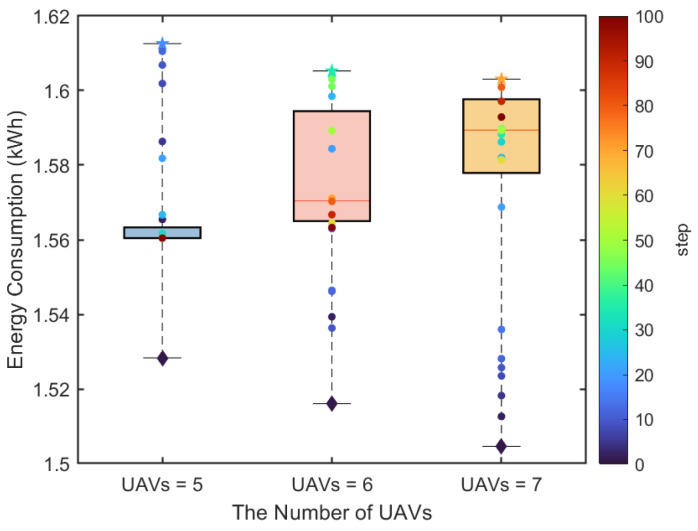
The USV energy consumption for different numbers of UAVs.

Figure 6 shows the change in USV energy consumption with the number of UAVs in the SCS algorithm. The discrete points represent energy consumption at different time steps, with diamond markers for the initial time step. It shows that the energy consumption of USVs initially increases and then decreases. Overall, in the initial stage, due to the random positions of UAVs and USVs as well as the random charging demands, the scheduling strategy needs to learn a better policy. Over time, USVs gradually optimize their movement direction through collaborative communication to minimize energy consumption while meeting the battery charging requirements of UAVs. Moreover, it can be seen that the energy consumption of USVs increases with the increasing number of UAVs because of serving more charging demands, requiring USVs to travel a longer distance and increasing energy expenditure.

**Figure 7:**
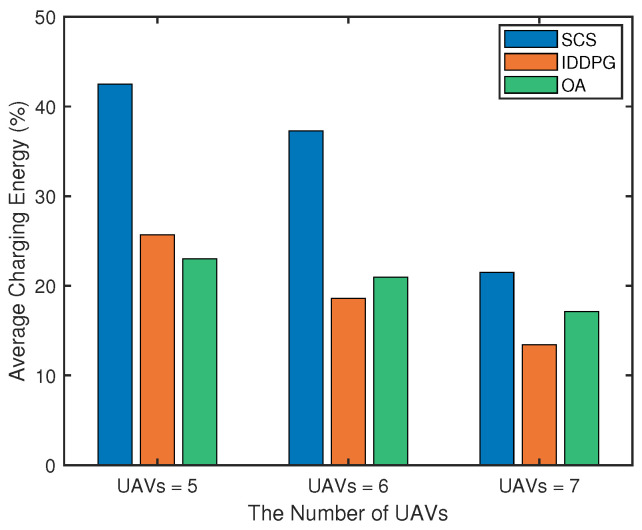
Comparison of average charging energy among different algorithms under different numbers of UAVs.

We further compare the average charging energy with different algorithms under varying number of UAVs. Average charging energy [43] refers to the average of the energy received by all UAVs in a single charging service. As illustrated in the Figure 7, the proposed SCS algorithm has significant advantages: when the number of UAVs increases from 5 to 7, the algorithm can keep the average charging energy at a high level. While the highest bidder gets the best bidding mechanism based on the online auction (OA) method [17] achieves a medium level, but its performance decreases significantly when the number of UAVs increases due to resource competition and suboptimal allocation in the bidding process. Compared with the independent deep deterministic policy gradient (IDDPG) [44], the independent decision mechanism may cause multiple USVs to repeatedly select an UAV service object, showing the lowest charging energy effect. With the expansion of the system scale, the SCS algorithm shows stronger adaptability, and the learned cooperative strategy can realize more reasonable path planning.

**Figure 8:**
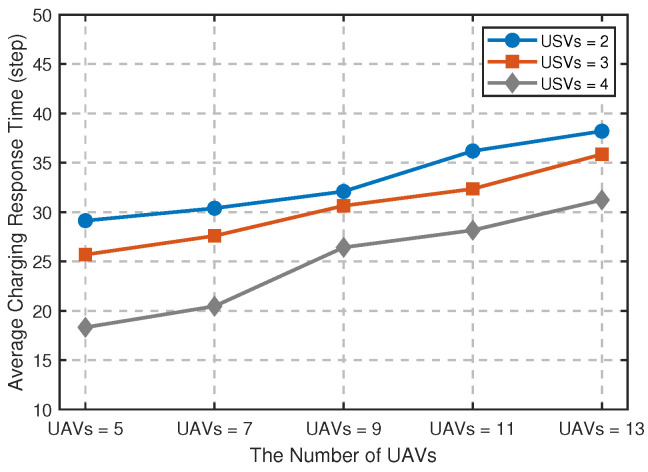
Comparison of average charging response time under different numbers of UAVs and USVs.

Figure 8 verifies the comparison results of average charging response time under different UAVs and USVs, where the response time is defined as the time interval from the UAV issuing the charging request to the actual charging start. The experimental results show that with the increase in the number of UAVs, the average charging response time of the system presents an upward trend, and there are obvious performance differences under different USV configurations. The higher the number of USVs, the shorter the response time, and the lower the number of USVs, the longer the response time, indicating that the number of USVs plays a key role in regulating the endurance capability of multi-UAVs. The data curve shows that as the number of UAVs increases to nine or more, the growth trend in system response time increases, indicating that there may be some reduction in synergy efficiency under the current configuration. This phenomenon suggests that under the existing system architecture, when the scale of UAVs reaches this critical value, it may be necessary to further optimize the resource scheduling strategy to maintain the ideal operational efficiency.

## 6. Conclusions

In this paper, we proposed a long-endurance collaborative SAR scheme leveraging the complementary advantages of UAV and USV, and deep reinforcement-learning techniques. This scheme includes a multi-UAV first-phase search algorithm and multi-USV second-phase search algorithm. In addition, to carry out long-endurance collaborative search, we designed a multi-USV mobile charging scheduling algorithm to prolong the flight time of UAVs. Numerical simulations are conducted to validate the effectiveness of the proposed scheme. Furthermore, for the operation of the UAV–USV search system in harsh environments, such as ocean currents or environments with variable communication requirements, the effectiveness of the proposed scheme should be further studied by adding more complex algorithms or methods. We leave it as our future research to enhance the robustness of the proposed scheme.

## Figures and Tables

**Table 1:** Simulation parameters.

Params	Description	Value (Unit)
$R_{CU}$	Communication distance of USVs	100 m
$D_{min}$	The safe distance between UAVs	3 m
$L_{min}$	The safe distance between USVs	5 m
$E_{max}$	Maximum energy capacity	97.58 Wh
$E_{th}$	Battery energy threshold	20%
*v*	UAV level speed	40 km/h
$\rho_{a}$	Air density	1.225 kg/m^3^
$A_{s}$	Total area of rotor disks	0.18 m^2^
$d_{0}$	Fuselage drag ratio	0.6
$S_{R}$	Rotor solidity	0.05 $m^{3}$
*d*	The detection probability	0.9
*f*	The false probability	0.1
*E*	Number of episodes	20,000
γ	Discount factor	0.95
α	Learning rate	0.01
τ	Target network update speed	0.01
*S*	Batch size	1024
$D,\tilde{D}$	Buffer length	$1\times 10^{6}$

**Table 2:** The uncertainty under the combination of different detection probabilities (*d*) and false probabilities (*f*).

	*f*	0.1	0.2	0.3	0.4	0.5
*d*						
0.5		0.832	0.860	0.944	0.984	1.000
0.6		0.690	0.776	0.866	0.950	0.984
0.7		0.583	0.650	0.786	0.866	0.929
0.8		0.451	0.531	0.668	0.736	0.854
0.9		0.305	0.390	0.507	0.587	0.664

## Data Availability

Dataset available on request from the authors.
